# Editorial: Non-cellular immunotherapies in pediatric malignancies

**DOI:** 10.3389/fimmu.2024.1379278

**Published:** 2024-02-21

**Authors:** Sarah Alexander, Paul Harker-Murray, Robert J. Hayashi

**Affiliations:** ^1^ Pediatrics, Division of Haematology/Oncology, Hospital for Sick Children, University of Toronto, Toronto, ON, Canada; ^2^ Pediatric Oncology, Medical College of Wisconsin, Milwaukee, WI, United States; ^3^ Pediatrics, Division of Pediatric Hematology/Oncology, Washington University School of Medicine, St. Louis, MO, United States

**Keywords:** immunotherapy, cancer, pediatrics, antibodies, checkpoint inhibition, vaccines

Advances in pediatric cancer treatment over the past three decades have focused on the intensification of conventional chemotherapy. While this approach was successful, we have exhausted this strategy for future progress in this field. Immunotherapy-using the immune system and its components to combat cancer has received considerable attention for many years with recent advancements elevating the enthusiasm for its potential to advance pediatric cancer care (1, 2). Substantial focus has been placed on cellular therapy, with promising results observed to date (3–6). While investigations in this area continue, there remains a vast array of other strategies in using elements of the immune system that warrants further attention.

The immune system has several components which can be harnessed to achieve anti-tumor responses. Broadly speaking, there are three fundamental strategies that encompass the scope by which non-cellular immunological strategies can combat cancer 1) Using the inherent specificity of the immune system to target specific tumor antigens 2) overriding inhibitory signals to the immune system imposed by the malignant cell, 3) augmenting tumor specific immune responses. Although each area warrants a thorough examination to fully assess its full potential, each varies in terms of both their flexibility and their promise for success.

## Using the specificity of the immune system to target cancer cells

Antigen specificity is central to why the immune system is attractive as a cancer treatment modality. Antigen specific antibodies have been developed, providing a variety of specifically targeted therapeutic agents. “Naked antibodies”-antibodies directed to tumor specific antigens were amongst the original agents developed, and several have had significant clinical impact in both pediatric and adult oncology care (7–9). There are probably several reasons why this strategy has not been even more successful: 1) Antibodies shown to kill tumors either via antibody-dependent cellular cytotoxicity or complement fixation in the laboratory may lack comparable activity in patients (10) 2) antigens can down regulate expression of a target reducing its accessibility (11)and 3) infused antibodies may be viewed as foreign antigens by the host and may be cleared by the host immune system (8, 12). Naked antibodies can have other anti-neoplastic properties by blocking a neoplastic cell’s biologic activity (13). Agents such as bevacizumab, an antibody against VEGF, had shown initial promise (14, 15), but targeted drugs against the VEGF signaling pathway may supplant antibody agent use as oral formulations are much easier to administer. Other targets may emerge as research efforts continue to identify accessible targets that are essential to cancer viability. Other strategies to use antibodies capitalize on their ability to target a tumor for delivery of anti-neoplastic agents. Antibody drug conjugates (ADC) have been particularly advantageous strategy as agents linked to chemotherapy agents have resulted in impressive responses in specific settings (16–18). The potential of this strategy is immense, and increasing our understanding of unique susceptibilities of specific tumors to specific agents may lead to a generation of new therapies that may even supplant conventional chemotherapy administration. Antibody linked radio-pharmaceuticals are currently in their infancy with regard to their use in children (19, 20). However, the ability to deliver therapeutic doses of radiation to targeted areas may allow for intensification of radiation dosing at a targeted site, sparing vital organs and thus long-term toxicity for a growing child. Finally, bispecific T cell engagers (BITE) bring the tumor targets in proximity to the host’s T cell, promoting cellular mediated cytotoxicity (21, 22). Down regulation of the targeted antigen and the possible up-regulation of checkpoint inhibition may limit its efficacy as agents are currently being developed across different tumor types.

## Overriding inhibitory signals to the immune system imposed by the malignant cell

The appreciation that checkpoint inhibition expression is a common means by which tumor cells escape immune surveillance was a major breakthrough in overcoming obstacles to eradicate cancer. The development of “checkpoint inhibitors “ agents, mostly antibodies, designed to target and block PD-1 or CTLA-4 have led to dramatic responses in a variety of adult malignancies which have historically been poorly controlled using conventional chemotherapy (23–25). The most successful agents are moving into the pediatric arena and defining the scope and management of toxicity and the efficacy for specific diseases will be the focus of many trials in the future.

## Augmenting tumor specific immune responses

In patients who possess an inherent ability to develop an immune response to a tumor target, efforts to enhance the response to optimize efficacy should be considered. Strategies have mostly focused on tumor “vaccines’ to prime the immune system to achieve an optimal response (26, 27). To date, there are few examples which have proven to be efficacious but numerous strategies are still being pursued including 1) searching for novel antigens (28) 2) loading dendritic cells to facilitate antigen presentation (29), 3) viral or molecular vectors expressing tumor antigens that will serve as targets for immune activation (30).

Cytokines, which were initially touted to be a source of anti-neoplastic therapy have failed to advance as a therapeutic agent. Further advancements in refinement in therapy may clarify the role of cytokines which will probably find its role in supportive care or enhancement of other elements of the immune response (31, 32).

In summary, in addition to the current excitement for the successes of cellular therapy, the scope of non-cellular immunotherapies will provide clinicians with new tools and strategies that may prove to be efficacious in cancer therapy. This Research Topic, *Non-cellular immunotherapies in pediatric malignancies* provides a view of the current efforts exploring how we can advance immunologic agents for pediatric cancer. As promising agents continue to emerge from adult clinical trials, efforts to not only assess an agent’s activity in pediatric malignancies are needed, but studies to refine dosing, schedules, and an agent’s use in combination with conventional chemotherapy or other biologic agents are needed. Coordinated efforts, particularly within large cooperative groups will facilitate rapid advancement of these novel agents so that their role in pediatric cancer therapy can be defined.

## Author contributions

RH: Writing – original draft, Writing – review & editing. SA: Writing – original draft, Writing – review & editing. PH: Writing – original draft, Writing – review & editing.
